# Mapping the Tooth Enamel Proteome and Amelogenin Phosphorylation Onto Mineralizing Porcine Tooth Crowns

**DOI:** 10.3389/fphys.2019.00925

**Published:** 2019-07-30

**Authors:** Daniel R. Green, Fabian Schulte, Kyu-Ha Lee, Megan K. Pugach, Markus Hardt, Felicitas B. Bidlack

**Affiliations:** ^1^The Forsyth Institute, Cambridge, MA, United States; ^2^Department of Human Evolutionary Biology, Harvard University, Cambridge, MA, United States; ^3^Department of Oral Health Policy and Epidemiology, Harvard School of Dental Medicine, Boston, MA, United States; ^4^Department of Developmental Biology, Harvard School of Dental Medicine, Boston, MA, United States

**Keywords:** tooth enamel, amelogenin, phosphorylation, posttranslational modification, enamel proteome, enamel mineralization

## Abstract

Tooth enamel forms in an ephemeral protein matrix where changes in protein abundance, composition and posttranslational modifications are critical to achieve healthy enamel properties. Amelogenin (AMELX) with its splice variants is the most abundant enamel matrix protein, with only one known phosphorylation site at serine 16 shown *in vitro* to be critical for regulating mineralization. The phosphorylated form of AMELX stabilizes amorphous calcium phosphate, while crystalline hydroxyapatite forms in the presence of the unphosphorylated protein. While AMELX regulates mineral transitions over space and time, it is unknown whether and when un-phosphorylated amelogenin occurs during enamel mineralization. This study aims to reveal the spatiotemporal distribution of the cleavage products of the most abundant AMLEX splice variants including the full length P173, the shorter leucine-rich amelogenin protein (LRAP), and the exon 4-containing P190 in forming enamel, all within the context of the changing enamel matrix proteome during mineralization. We microsampled permanent pig molars, capturing known stages of enamel formation from both crown surface and inner enamel. Nano-LC-MS/MS proteomic analyses after tryptic digestion rendered more than 500 unique protein identifications in enamel, dentin, and bone. We mapped collagens, keratins, and proteolytic enzymes (CTSL, MMP2, MMP10) and determined distributions of P173, LRAP, and P190 products, the enamel proteins enamelin (ENAM) and ameloblastin (AMBN), and matrix-metalloprotease-20 (MMP20) and kallikrein-4 (KLK4). All enamel proteins and KLK4 were near-exclusive to enamel and in excellent agreement with published abundance levels. Phosphorylated P173 and LRAP products decreased in abundance from recently deposited matrix toward older enamel, mirrored by increasing abundances of testicular acid phosphatase (ACPT). Our results showed that hierarchical clustering analysis of secretory enamel links closely matching distributions of unphosphorylated P173 and LRAP products with ACPT and non-traditional amelogenesis proteins, many associated with enamel defects. We report higher protein diversity than previously published and Gene Ontology (GO)-defined protein functions related to the regulation of mineral formation in secretory enamel (e.g., casein α-S1, CSN1S1), immune response in erupted enamel (e.g., peptidoglycan recognition protein, PGRP), and phosphorylation. This study presents a novel approach to characterize and study functional relationships through spatiotemporal mapping of the ephemeral extracellular matrix proteome.

## Introduction

The strength and stiffness of tooth enamel resemble the properties of some metal alloys and are achieved through the hierarchical arrangement of hydroxyapatite (HAp) mineral crystals within a proteinaceous matrix (Bechtle et al., 2012; Wegst et al., 2015; Yilmaz et al., 2015). The three structural proteins amelogenin (AMELX), enamelin (ENAM), and ameloblastin (AMBN), and the two matrix proteases matrix-metalloproteinase 20 (MMP20) and kallikrein-related peptidase 4 (KLK4) are well studied, and their posttranslational modifications impact HAp formation and enamel health (Chun et al., 2010; Wiedemann-Bidlack et al., 2011; Yamakoshi, 2011; Bartlett, 2013; Hu et al., 2014; Lacruz et al., 2017; Yamazaki et al., 2017; Yan et al., 2017; Le Norcy et al., 2018). The spatial organization and timing of protein deposition, posttranslational modification and removal is tightly regulated to guide mineralization. Yet, the spatiotemporal pattern of relative protein abundance and proteolytic processing, as well as posttranslational modification status including phosphorylation is not fully resolved for the three classic enamel matrix proteins AMELX, ENAM, AMBN, and their cleavage products, and is less resolved for proteins during this process (Uchida et al., 1991, 1997; Tanabe et al., 1992; Moradian-Oldak, 2012; Bartlett, 2013; Gallon et al., 2013; Mazumder et al., 2016; Yamazaki et al., 2017).

Ameloblasts secrete AMELX, AMBN and ENAM, which are structural proteins unique to enamel and derived from the secreted calcium-binding phosphoprotein (SCPP) gene family that evolved over 600 million years ago (Kawasaki and Weiss, 2003; Kawasaki and Weiss, 2006, 2008). AMELX is the most abundant enamel matrix protein with only one described posttranslational modification, a phosphorylation at serine 16, which is likely to be also present in the amelogenin splice variant P190 (27 kDa) localized at very low abundances at the enamel surface (Takagi et al., 1984; Fincham et al., 1994; Yamakoshi et al., 1994, 2006a; Fincham and Moradian-Oldak, 1995; Yamakoshi, 2011). Serine 16 is known to be phosphorylated in P173 cleavage products and in the second-most abundant isoform LRAP which consists of the N- and C-terminal domains of P173 (Fincham et al., 1994; Yamakoshi et al., 1994, 2011; Fincham and Moradian-Oldak, 1995; Nagano et al., 2009). Phosphorylated AMELX and its cleavage product P148 that accumulates in pig enamel have been shown to stabilize amorphous calcium phosphate *in vitro* for longer time than their unphosphorylated forms (Wiedemann-Bidlack et al., 2011). LRAP phosphorylation appears to affect mineralization activity of ameloblast cell lines and cultured tooth germs (Wiedemann-Bidlack et al., 2011; Le Norcy et al., 2018). Studies in transgenic mouse models have shown that the presence and relative abundance of amelogenin N-terminal, C-terminal, and hydrophilic core domains are important for the generation of proper tooth enamel (Xia et al., 2016; Bidlack et al., 2017). AMELX, ENAM and AMBN are cleaved by MMP20 upon secretion and by KLK4 during later stages of enamel formation (Yamakoshi et al., 2006a, 2011; Nagano et al., 2009; Chun et al., 2010). All three matrix proteins are crucial for healthy enamel formation and their orchestrated removal is critical to allow for expansion of the mineral phase and completion of enamel mineralization (Simmer and Hu, 2002; Chun et al., 2010; Yamakoshi et al., 2011; Hu et al., 2016; Lacruz et al., 2017). No enamel is formed in mice without phosphorylation of ENAM or AMBN (Chan et al., 2010; Ma et al., 2016; Yan et al., 2017).

Changes in AMELX, ENAM, and AMBN abundance and modification occur within a broader proteomic context that helps regulate mineralization. Proteomic studies of enamel, dentin, bone and cementum have identified over 200 unique proteins (Eckhardt et al., 2014; Jagr et al., 2014; Charone et al., 2016; Pandya et al., 2017; De Lima Leite et al., 2018) with functions that include calcium binding, cytoskeletal and cell adhesion, immune function, proteolysis and protease inhibition. Gel electrophoresis and nano-LC-MS/MS analyses identified structural molecules including 20 keratins, collagens, serpins, ubiquitin and serum albumin (Webb et al., 1998; Felszeghy et al., 2000; Acil et al., 2005; Yamakoshi et al., 2006b;Jagr et al., 2012; Salmon et al., 2013; Castiblanco et al., 2015). Messenger RNA transcript profiling studies have identified as many as 1700 gene transcripts that are upregulated or downregulated in ameloblasts during either early or late phases of enamel formation (secretion or maturation stage) with annotations in the Gene Ontology (GO) database corresponding to ion transport, pH regulation, calcium interactions, and other functions (Lacruz et al., 2011, 2012; Yin et al., 2014).

Though recent proteomics analyses of human and murine teeth indicate a high diversity of extracellular proteins during enamel formation, results so far have focused on the analysis of either entire molars, or of secretion and maturation stages of enamel formation in incisors, or have targeted the dentin-enamel junction, but have not addressed spatiotemporal abundance levels in more detail (Castiblanco et al., 2015; Charone et al., 2016; Pandya et al., 2017; De Lima Leite et al., 2018; Jágr et al., 2019). This is in part due to the small size of mouse teeth impeding the separation of enamel from dentin and pulp. In the present study, we have aimed to overcome these hurdles by using larger porcine molars that allowed us to microsample across all stages of enamel formation and to compare enamel with dentin and bone. Microsampling in a grid pattern, we have sought to (a) spatiotemporally map the proteome onto the developing enamel and erupted tooth crown, (b) identify new players in enamel amelogenesis, and (c) leverage our proteomic approach to identify specific splice and cleavage products, as well as posttranslational modifications of amelogenin at specific stages and locations of enamel mineralization.

## Materials and Methods

### Tooth Collection and Enamel Dicing

**FIGURE 1:**
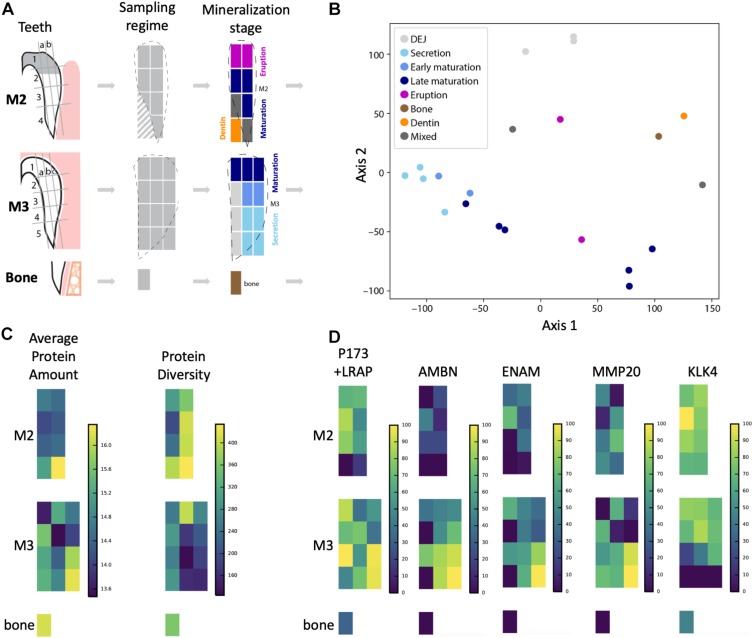
Overview of sampling strategy and stages of enamel mineralization in the forming tooth crown. **(A)** Porcine molars that were partially erupted (M2) or unerupted and still in the process of crown extension (M3) were dissected and enamel diced into sample blocks, dentin and bone sampled for comparison, and proteins extracted for LC-MS/MS analysis. Dotted lines show outlines of enamel edges relative to diced sample blocks, which are color-coded according to *a priori* anatomical estimates of tissue and mineralization stage. Diagonal hashes show where dentin tissue was sampled. **(B)** Principal components analysis shows that samples from secretory enamel sample locations cluster tightly together (light blue), while early and late maturation (darker blue) cluster more loosely. Bone (brown) and dentin (orange) locations cluster separately, as do DEJ samples (light gray). **(C)** Average peptide amount (peak area) is highest in secretory enamel, while peptide diversity is higher in maturation and erupted enamel. High protein abundance is shown by bright yellow coloration and low abundance by dark blue on the left. High protein diversity is shown by bright yellow and low diversity by dark blue on the right. **(D)** Abundance for the five traditional amelogenesis proteins, measured as the natural log of the peptide LC-MS/MS peak area and shown on a percent (0–100%) scale per protein, where bright yellow corresponds to high, and dark blue corresponds to low abundance. As expected based on previous observations for these proteins, we observe that AMELX, AMBN, ENAM, and MMP20 have highest abundances in secretory enamel, whereas we do not observe KLK4 in secretory enamel and instead find it in maturing and mature enamel. The spatial pattern seen for the amelogenin P173+LRAP is more complex because we simultaneously track all amelogenin P173+LRAP derived proteins exhibiting the N-terminal sequence (e.g., P173, P56, and their N-terminal derived processing products including P162, P148, P62/3, P45 or TRAP, LRAP, and P40).

All samples were collected from a euthanized 8-month-old female pig in agreement with IACUC regulations of the Tufts University Cummings School of Veterinary Medicine and The Forsyth Institute. Mandibles were dissected out, immediately transferred to dry ice for transport and stored at –80^∘^C until further processing. Erupted and unerupted mandibular molars were excavated and extracted on ice with a Dremel saw, soft tissue and adhering cells pulled off. Coronal sections of 1.2 mm thickness were cut under a continuous, non-recycled stream of 1% PBS from the second (M2) and third (M3) molars using a low-speed saw (Isomet, Buehler, Lake Bluff, IL, United States) with a diamond blade (diamond wafering blade 102 × 0.3 mm, Buehler, Lake Bluff, IL, United States). Sections passed through dentin horns and maximum cervical enamel extent on buccal and lingual sides. After cutting, the tooth crown surface was lightly scraped with a scalpel blade to remove debris. Each section was washed three times under continuous 1% PBS rinse for 30 s and mounted onto silica wafers with Quickstick 135 Mounting Wax Electron Microscopy Sciences, Hatfield, PA, United States). The enamel from each section was diced in a grid pattern at the Massachusetts Institute of Technology Media Technology Laboratories using a DAD3400 saw with zinc aluminum blades (0.03 mm; Z09–SD1700–Y1–60, Disco Hi-Tech America) under continuous flow of distilled water. Resulting enamel and dentin blocks (*n* = 20) were 3 mm long, 0.5–0.6 mm wide and 1.2 mm thick (Figure 1A). Mean block volume was 2.3 mm^3^. Variations in volume resulting from adjustments made in sections close to the enamel dentine junction and crown morphology were quantified using imaging analyses of the surface area in FIJI^1^. Each enamel block was taken up from the wafer by forceps under a microscope, placed in a separate 2 mL microcentrifuge tube, and gently rinsed five times with 1 mL 1% PBS solution. A sample piece of alveolar bone adjacent to the M2 was removed by forceps and processed in the same manner as enamel samples.

### Protein Extraction

Each enamel block was dissolved in 1 mL of 12% trichloroacetic acid under agitation at 450 RPM for 48 h at room temperature (RT), then centrifuged at 4^∘^C for 45 min (2,500 × *g*), and the supernatants discarded. Pellets were twice washed with 200 μL acetone, centrifuged at 13,000 × *g* for 10 min at RT, supernatants discarded, samples evaporated for 1 h at RT and pellets resuspended in 20 μL of 8 M urea followed by 55 μL of 50 nM ammonium bicarbonate (ABC) buffer solution. Proteins were reduced by the addition of 6 μL 1 M dithiothreitol (DTT) in ABC for 15 min in the dark at RT, then alkylated with 30 μL 200 μM iodoacetamide (IAA) in ABC for 30 min at 37^∘^C, and the reaction quenched with 12 μL DTT. Proteins were digested by the addition of 0.2 μg/μl trypsin (Trypsin Gold, Promega) and incubation at 37^∘^C for 48 h. Digestions were stopped by the addition of 5 μl 10% TFA, and the digests were desalted over Pierce C18 tips and resuspended in 24 μL 0.1% formic acid after solvent evaporation in a SpeedVac. For normalization of nLC-chromatogram retention times, we added custom iRT peptide standard variants to each sample for a final concentration of 50 nM (Escher et al., 2012).

### Nano-Liquid Chromatography Electrospray Ionization Tandem Mass Spectrometry (Nano-LC-ESI-MS/MS)

A total of 5 μL of each sample was injected, in randomized order, into an EASY-nLC 1000 liquid chromatography unit (Thermo Scientific) hyphenated to a QExactive Plus mass spectrometer (Thermo Scientific). Samples were loaded onto the trap column (Acclaim PepMap 100, 100 μm × 2 cm) for 9 min at 2 μl/min in a mixture of mobile phase buffer A (0.1% formic acid in water) and mobile phase buffer B (0.1% formic acid in acetonitrile) and subsequently separated on a 50 cm EASY-Spray column (ES803, 75 μm × 50 cm, C18, 2 μm, 100 Å). Mobile phase B increased from 2 to 32% at 150 min at a flowrate of 200 nL/min followed by a 30-min wash at 72% and a 20-min re-equilibration at 0% B. Data was acquired in positive ionization mode using data-dependent acquisition (DDA) with the following parameters: Precursor ion survey scans from 350 to 1200 m/z were acquired at 70k resolution (at 200 m/z) with an automatic gain control (AGC) target of 3.0 × 10^6^ ion counts. Subsequent MS/MS analyses were conducted using the Top20 approach. The most abundant precursors (intensity greater than 2.0 × 10^4^ ions) were isolated in the quadrupole mass analyzer using a 2.5-Da m/z isolation window and fragmented by higher-energy collisional dissociation (HCD) using a normalized collision energy of 28. Fragment ions were detected in the Orbitrap mass analyzer with a resolution setting of 17,500, an AGC target setting 1 × 10^5^ and a maximum ion accumulation time of 150 ms. Previously analyzed precursor ions and isotopes were dynamically excluded for 40 s. Only precursors with assigned charge states larger than 1 were selected for MS/MS.

### Protein Identification

Protein identification was performed by using the PEAKS Studio software suite (version 8.0) (Zhang et al., 2012). Raw instrument data was refined using the following parameters: scans were merged over 10 min retention time windows using a 10-ppm precursor m/z error tolerance; precursor mass and charge states (*z* = 1–10) were corrected. Other data pre-processing (centroiding; deisotoping; deconvolution) was performed automatically. Feature detection was performed on each raw instrument file individually. PEAKS database searching was performed against the UniprotKB database for *Sus scrofa* (Pig) containing 34,524 entries (downloaded September 2017). Search parameters were: parent mass error tolerance 10 ppm; fragment mass error tolerance 0.05 Da; trypsin enzyme specificity with cleavage prior to proline permitted; variable modifications: pyro-glu (Q), oxidation (M), deamidation (NQ), HexNAcylation (N), and phosphorylation (STY); maximal two variable modifications per peptide; one non-specific cleavage specificity on either terminus, maximal two missed cleavages, maximal two variable posttranslational modifications per peptide. The false discovery rate was estimated using the decoy-fusion approach (1% on the PSM-level). Proteins with at least two unique peptides were considered identified. Protein significance threshold was set at 20 (-10lgP), where higher significance values indicate greater confidence in the identification of peptides (Tran et al., 2019).

Protein abundances were based upon area-under-the-curve measurements for the three most abundant unique peptides ascribed to that protein. Abundances were subsequently normalized to microsampling volumes compared to a reference standard sample volume of 2.14 mm^3^ to account for variation in sample volume, for example in regions of thinning enamel at the cervical margin (Supplementary Table 1). For the visualization of each peptide, abundance was shown on a percent scale normalized to the peptide’s highest abundance in the tooth crown (Supplementary Table 1). The underlying raw proteomics data has been made publicly available through the PRIDE repository.

Peptides (LPPHPGHPGYINF**S**YEK, P190; MPLPPHPG HPGYINF**S**YEVLTPLK, P173, LRAP) were used to discern the phosphorylated and unphosphorylated variants of Ser16 of the parent molecules and derived cleavage products of P190 on the one hand, and P173 and/or LRAP on the other hand. These N-terminal peptide fragments permit identification of the parent molecules P190, P173, and LRAP, but do not necessarily allow distinction between all cleavage products resulting from these original molecules. We used the ameloblastin peptide QPGTPGVASL**S**LETMR that is consistent with both AMBN 15 and 17 kDa cleavage products to map phosphorylated and unphosphorylated AMBN. For mapping phosphorylated and unphosphorylated enamelin, we used the peptide GYHGFGGRPPYY**S**EEMFEQDFEKPK, which is representative of the 89, 142, 155, and 32 kDa cleavage products.

**FIGURE 2:**
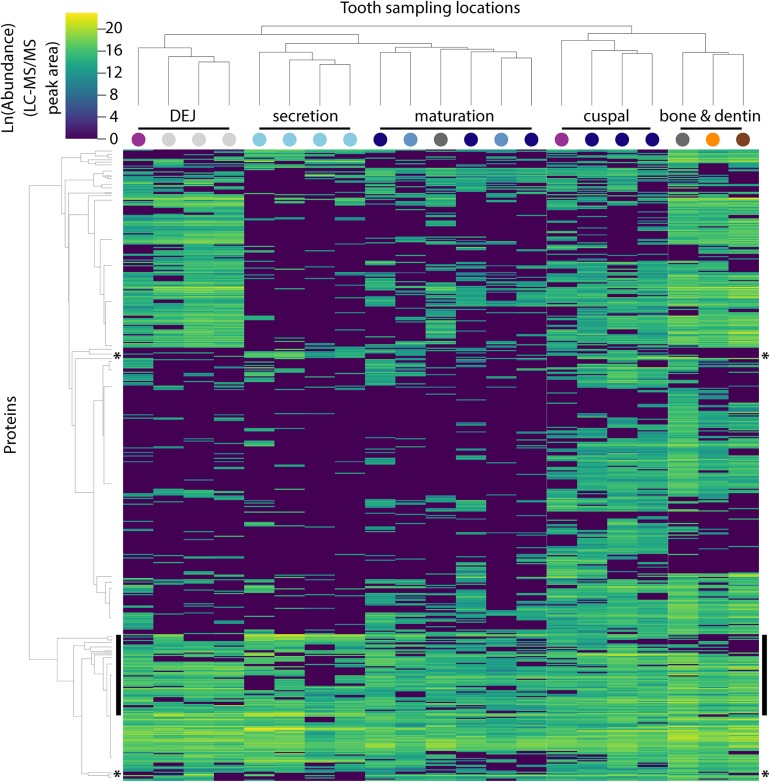
Hierarchical clustering of peptides and sample locations based upon abundance patterns. Overview of all 551 peptides and 21 sample locations clustered by Euclidian distance, showing that secretory proteins are tightly clustered compared to other sampled locations, a result consistent with our PCA (Figure 1B). Sample location clustering compared to *a priori* anatomical assignment indicated by colored circles (see Figure 1). Most known amelogenesis proteins including P173+LRAP products, AMBN, ENAM and MMP20 cluster tightly together (vertical black bars); stars indicate the location of the P190 AMELX isoform (above) and KLK4 (below).

**FIGURE 3:**
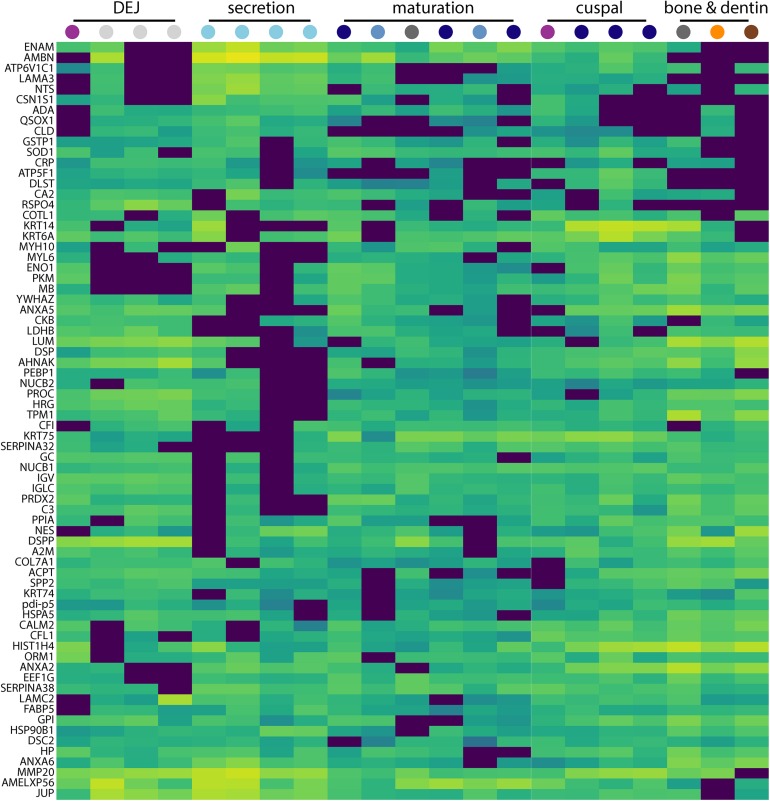
Secretion-specific hierarchical clustering of peptides. A portion of the data shown in Figure 2 is enlarged here to provide a more detailed view of the region of hierarchical clustering showing traditional amelogenesis proteins ENAM, AMBN, P173+LRAP, and MMP20 alongside others with similar abundance patterns including CSN1S1, ACPT, LAMA3, NTS, and COL7A1. Figure scale and legend is the same as that shown in Figure 2. Many proteins that localize to this region of the hierarchical clustering analysis are associated with enamel defects when their associated genes are mutated or absent in humans and rodents.

PCA (Figure 1B) and hierarchical clustering (Figures 2, 3) were performed with the Scikit-learn module in Python 3.6. Data visualization was provided by the Python matplotlib and seaborne modules. Gene Ontology (GO) classification enrichment analyses (Supplementary Table 1 and Supplementary Figure 1) were conducted using the DAVID 6.8 online Bioinformatics Resource with default stringency criteria, employing the whole pig proteome as a reference database. Enrichment analyses were conducted for proteins found in each of the four Euclidean distance enamel location clusters shown in Figures 2, 3, excluding the lowest 10th percentile least abundant proteins in each group. To locate those proteins with differential abundance in one location cluster compared to others, we conducted a non-parametric ANOVA using permutation tests for each protein (Anderson, 2001) and performed the Benjamini–Hochberg procedure to account for multiple comparisons (Benjamini and Hochberg, 1995). We then observed cluster means to determine whether proteins identified as having differential abundance more or less abundant in each cluster (Supplementary Table 1 and Supplementary Figure 1).

## Results

We identified 551 unique UniProtKB protein entries collectively in molar porcine enamel samples (SI 01). Of the proteins detected in enamel, 211 were within middle enamel, that is sample blocks not adjacent to the crown surface, or the dentine-enamel interface. Of all proteins identified, 84% had Gene Ontology (GO) annotations; 66% of these were annotated as extracellular, and 36% as cytosolic, with overlap expected between these groups (e.g., actin, S100, annexin; Supplementary Table 1). Average protein coverage in enamel and dentin samples was 50%. We show spatial co-occurrence of proteins pertaining to key steps during enamel secretion and maturation, and follow the spatial abundance P190 and P173+LRAP and their phosphorylation status on serine 16.

The approach from enamel micro-sampling strategy to localizing proteins and mapping their distribution during enamel development is summarized in Figure 1. The unerupted third molar included the enamel formation stages of secretion, and early and late maturation, while the partially erupted second molar allowed us to explore posteruptive enamel changes through the comparison with mature enamel of the not yet erupted part of the same tooth (Figure 1A). A principal components analysis of the spatial pattern of abundance of all 551 identified proteins showed that in the first and second PCA axes, secretory and early maturation locations cluster tightly together compared to later maturation (Figure 1B). In these axes bone and dentin data segregate from enamel samples that were clearly associated with secretion and maturation, while data from erupted enamel sorted with both maturation stage and with locations that include a mix of enamel formation stages and tissues. Protein diversity was lowest during secretion and highest in late maturation stage enamel (Figure 1C). By contrast, average protein abundance was highest in secretory and early maturation stage enamel. Hierarchical clustering of protein abundance similarity and sample location using Euclidian distance demonstrates which proteins are found in high abundances together in specific regions of the developing enamel. This analysis revealed five clear groupings (Figure 2). Enamel samples from the secretory stage clustered most closely together. Although maturation stage locations also clustered with secretory stage, abundance data were slightly more variable in maturation compared to secretory enamel. Enamel adjacent to the dentin-enamel junction (DEJ) shared these proteins but added a third cluster, while a fourth cluster was found in bone and dentin and a fifth in cuspal enamel. Most known amelogenesis proteins including ENAM, AMBN, MMP20, LRAP, and carbonic anhydrase 2 (CA2) clustered tightly together on the protein dendrogram, as expected (Figure 3).

**FIGURE 4:**
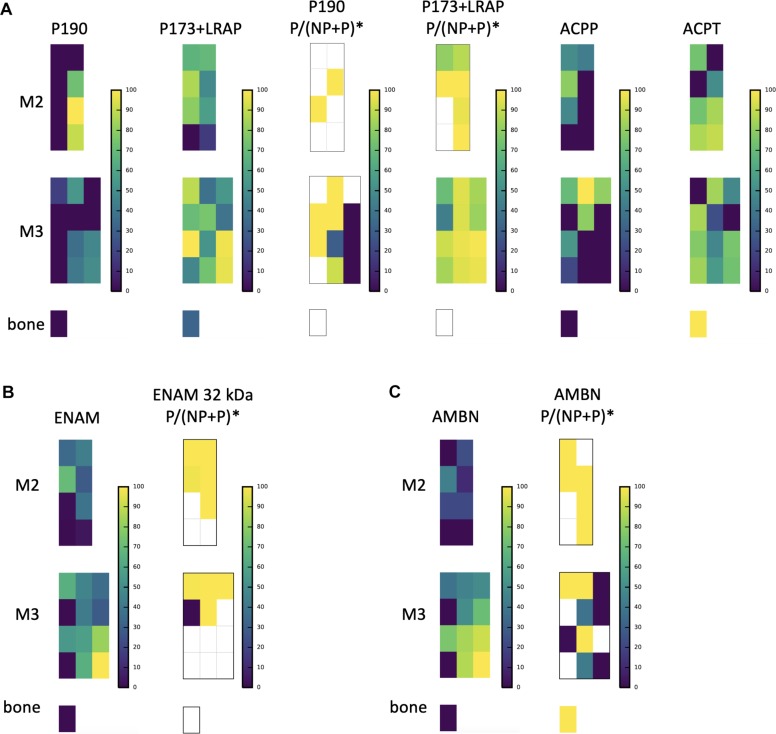
A subsample of protein abundance and posttranslational modification maps in forming teeth. **(A)** P190 and P173+LRAP abundance distributions are shown on the left, followed by the ratio of phosphorylated form to phosphorylated plus non-phosphorylated (P:P+NP) peptides, and on the right abundance distributions of testicular acid phosphatase precursor (ACPP) and protein (ACPT), which have dephosphorylating functions and are associated with enamel defects when mutated. **(B)** ENAM distribution shown next to the distribution of the ratio of the phosphorylated to phosphorylated plus non-phosphorylated (P:P+NP) ENAM peptide corresponding to the 32 kDa cleavage product. Our results show that the peptide is present primarily in more mature enamel, consistent with its resistance to MMP20 proteolytic processing, and that it is almost wholly phosphorylated (one unphosphorylated location corresponds to very low abundance overall). **(C)** AMBN overall abundance followed by the ratio of phosphorylated AMBN. ^*^The ratio of the phosphorylated peptide to total peptide abundance normalized over the entire enamel area analyzed; white space indicates no peptide data at this location.

Amelogenin, AMBN, ENAM, and MMP20 proteins located to forming and erupting enamel, with highest protein concentrations in secretory and early maturation enamel (Figure 1D and Supplementary Table 1, for all 551 proteins). Higher abundance during secretory-stage was especially pronounced for AMBN, while residual quantities of AMELX and MMP20 were detected in maturing enamel. In contrast, KLK4 was not detected in secretory stage enamel, but highly abundant in early and late maturation stage in both molars. Consistent with prior research, we saw small quantities of AMELX and KLK4 in bone (Haze et al., 2007; Ramsay et al., 2008; Jacques et al., 2014). Products of the amelogenin splice variant P190 (which includes exon 4) were restricted near the crown surface and within forming enamel, whereas P173 and LRAP products were found throughout the enamel, with highest abundance levels in secretory and early maturation stage enamel (Figure 4A).

The relative abundance of phosphorylated product fragments derived from P173 and LRAP was higher near the crown surface in secretory and early maturation stage enamel. Notably, the distribution of the phosphatase ACPT was observed from the enamel surface into the middle of enamel and toward the DEJ, like AMELX and other enamel proteins (Figure 4A). The percent abundance of the phosphorylated ameloblastin N-terminal fragment was low in immature and superficial enamel, and higher in deeper and more mature enamel (Figure 4B) compared to the unphosphorylated form. Enamelin fragments analyzed for phosphorylation status showed minimal abundance of unphosphorylated forms in nearly all analyzed samples (Figure 4C).

**FIGURE 5:**
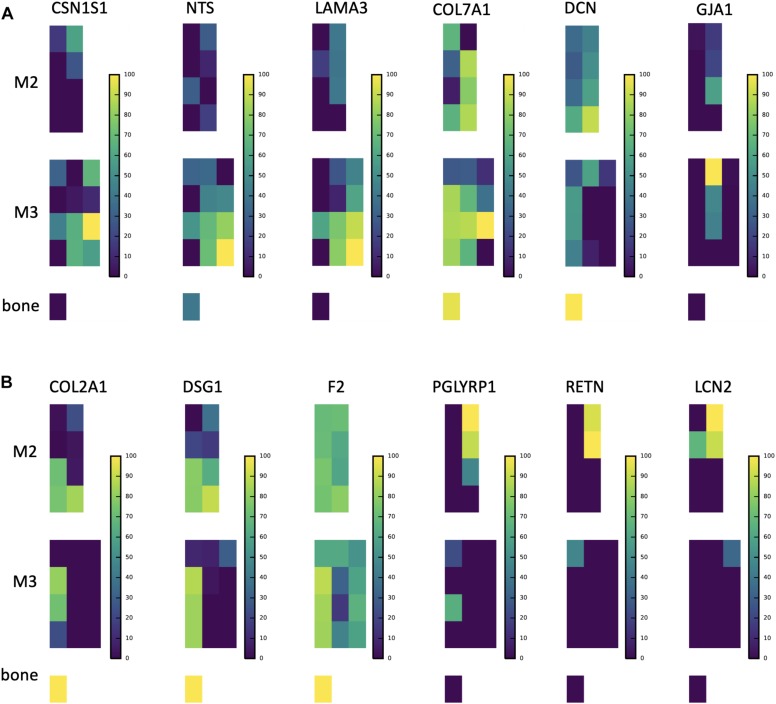
Abundance distributions of other notable proteins. **(A)** CSN1S1 is known to stabilize amorphous calcium phosphate minerals and is a member of the secreted calcium-binding phosphoprotein (SCPP) gene family, but is not traditionally found in enamel. We observe it in secretory enamel alongside proteins (NTS, LAMA3, COL7A1) known to be associated with enamel defects. We show DCN and GJA1, also known to be associated with enamel defects but found outside the secretory region. **(B)** Proteins that we find at high abundance at the DEJ (COL2A1, DSG1, F2), a result consistent with a recent proteomic analysis of the DEJ in humans, and proteins that include immune functions and are known to be produced by porcine salivary glands (PGLYRP1, RETN, LCN2). We find these at high abundances within erupted enamel. Peptide maps are shown on a percent (0–100%) scale per peptide. For abundances of all 551 proteins consult SI Supplementary Figure 1.

Interestingly, in secretory enamel we found alpha-s1-casein (CSN1S1), a phosphoprotein typically associated with milk, and that is also derived from the SCPP family. CSN1S1 is known to stabilize amorphous calcium phosphate and prevent the formation of crystalline mineral from a solution supersaturated with respect to calcium and phosphate (Figure 5A; Cross et al., 2004, 2016; Kawasaki et al., 2011). While to our knowledge CSN1S1 has not been previously located in forming enamel, numerous studies have used CSN1S1 for biomimetic enamel remineralization (Cross et al., 2004, 2016; Baroni and Marchionni, 2011; Mastroberardino et al., 2012). We found the CSN1S1 distribution to coincide with secretory enamel regions where amorphous calcium phosphate has been reported in murine enamel (Beniash et al., 2009).

In secretory enamel we detected higher abundances of collagen VI, gelsolin, annexin, the cell adhesion molecule junction plakoglobin, V-Type proton ATPases, the protease inhibitor anti-thrombin, and neurotensin precursor (Figure 5A and Supplementary Table 1). Collagen VII and carbonic anhydrase 2 were detected in both secretory and maturation stage enamel. Relative to other stages, mature enamel contained more V-Type ATPase H+ transporting subunits including B2, E1, and H, and also expressed the metabolic enzymes pyruvate kinase and phosphoglycerate kinase. In erupted enamel, we detected higher quantities of lipocalin 2 (LCN2), resistin (RETN), and peptidoglycan-recognition protein 1 (PGLYRP1) (Figure 5B), all of which were previously identified in saliva and are known to be involved in innate and adaptive immunity (Mamali et al., 2012; Aqrawi et al., 2017; Nylund et al., 2017; Tavladaki et al., 2017; Xiao et al., 2017; Dziarski and Gupta, 2018; Prims et al., 2019). Proteins we located at high abundance at the DEJ include desmoglein-1 (DSG1), collagen alpha-1(II) chain (COL2A1), and fibronectin (FN), consistent with recently published results obtained through a laser-based microsampling and tandem MS technique (Jágr et al., 2019).

Within each of four hierarchical cluster-defined groups of enamel samples (DEJ, Secretion, Maturation and Cuspal, Figure 2), we averaged protein abundance measurements and conducted functional enrichment analyses for each group, ignoring the lowest 10th percentile of proteins to avoid noise (SI 04). Annotations enriched compared to a whole pig proteome reference were similar for each group, with extracellular exosome, phosphoprotein, acetylation, glycoprotein, signal, and disulfide bond annotations among the top 15 statistically significant results for each.

Non-parametric tests located proteins with statistically significant increases or decreases in abundance across each group, with most significant results shown in Table 1. These tests revealed 99 proteins with decreased abundance in secretion compared to other enamel stages (Group 1), consistent with the observation that protein diversity is lowest in secretory enamel (Figures 1C, 2). Similarly, we found 68 proteins with decreased abundance in secretion and maturation relative to other enamel formation stages (Group 2). By contrast proteins with increased abundance in secretory enamel relative to the DEJ and maturation (Group 3) were few, with only 20 proteins located as significant but including MMP20 and the AMELX splice variant P190. Full lists are shown in Supplementary Figure 1.

**TABLE 1:** Ten proteins with the most statistically significant changes in abundance across DEJ, secretion, maturation, and cuspal enamel locations.

**Group 1**		**Group 2**		**Group 3**		**Group 4**	
			
**Protein**	***p*-value**	**Protein**	***p*-value**	**Protein**	***p*-value**	**Protein**	***p*-value**
Nucleobindin 1	<0.0001	Osteoglycin mimecan	<0.0001	40S ribosomal protein S18	0.0006	C-type lectin domain containing 11A	<0.0001
V-type proton ATPase subunit a	<0.0001	Procollagen-proline 2-oxoglutarate-4-dioxygenase	<0.0001	V-type proton ATPase subunit S1	0.0008	Transketolase	0.0006
Catenin delta 1	<0.0001	Solute carrier family 25 member 3	<0.0001	Ribosomal protein L15	0.0010	Transforming growth factor beta-1	0.0010
Galectin	<0.0001	Heat shock protein family A (Hsp70) member 9	<0.0001	40S ribosomal protein S4	0.0016	Transforming growth factor beta-2	0.0014
Myosin heavy chain 14	<0.0001	Semaphorin 3E	<0.0001	Matrix metalloproteinase-20	0.0018	IgG heavy chain	0.0016
S100 calcium binding protein A4 (Fragment)	<0.0001	Elastin microfibril interfacer 1	<0.0001	fibrinogen alpha chain	0.0022	Complement C3	0.0024
Protein S100-A6	<0.0001	Plastin 1	<0.0001	Myosin heavy chain 14	0.0024	Secernin 1	0.0040
Integrin alpha V (Fragment)	0.0002	Heterochromatin protein 1 binding protein 3	<0.0001	Heterogeneous nuclear ribonucleoprotein A3	0.0030	Apolipoprotein E	0.0042
Serpin family D member 1	0.0002	Ribosome binding protein 1	<0.0001	Junction plakoglobin	0.0032	Chitinase domain containing 1	0.0042
Coagulation factor X	0.0002	N-myosin-9	<0.0001	Keratin 13	0.0038	Olfactomedin like 3	0.0070

*Group 1 proteins are those with decreased abundance in secretion compared to other enamel stages, Group 2 proteins are those with decreased abundance in both secretion and maturation regions compared to DEJ or erupted regions. These groups are larger than other groups, consistent with the observation that protein diversity is lowest in secretory stage, and then increases in maturation enamel (Figures 1C, 2). By contrast, proteins with increased abundance in secretory enamel relative to the DEJ and maturation (Group 3) were few, with only 20 proteins located as significant but including MMP20 and the AMELX splice variant P190. Group 4 includes proteins with high abundance at the DEJ and in maturation stage. For the full lists of proteins, see Supplementary Table 1 and Supplementary Figure 1.*

## Discussion

### Protein Mapping and Sourcing

The sampling and analytical approach presented here provides a methodology to characterizing the spatiotemporal pattern of enamel formation based on proteomic data. The spatial distributions of AMELX, AMBN, ENAM, MMP20, and KLK4 (Figures 1D, 4) agree very well with published data on abundance patterns during specific stages of enamel formation (see Moradian-Oldak, 2012; Bartlett, 2013; Lacruz et al., 2017 and references therein). Our findings are consistent with the number and identifications of proteins of recently published proteomics analyses of pooled human and rodent enamel (Chen et al., 1995; Hubbard and Kon, 2002; Jagr et al., 2012, 2014; Eckhardt et al., 2014; Charone et al., 2016; Pandya et al., 2017; De Lima Leite et al., 2018; Mann et al., 2018; Zhang et al., 2018). For instance, we mapped 19 of the 24 proteins recently reported in enamel onto enamel formation stages (Pandya et al., 2017), and localized close homologs for the remaining five proteins (Supplementary Table 1). Our spatial proteomic data for AMBN, ENAM, MMP20, and CA2 conform nearly identically to the abundances observed through western blotting of successive stages of mouse molar enamel mineralization as reported by Pandya et al. (2017). The distribution we found for AMELX slightly deviates from results reported in Pandya et al. (2017), likely due to differences in the specific target peptide analyzed.

In agreement with the findings of the recent study by (De Lima Leite et al., 2018), we locate AMELX, ENAM, AMBN, MMP20, and other proteins thought to belong to the enamel matrix including α-2-HS-glycoprotein (AHSG), matrix Gla protein (MGP), and serpinh1 (SERPINH1) within the mineralizing portion of incisal enamel. In addition, we detect KLK4 and amelotin (AMTN), which De Lima Leite et al. (2018) expect but do not report in their dataset (Figure 1D and Supplementary Table 1). The recent analyses presented by (Jágr et al., 2019) located 49 proteins in dental enamel, and 15 proteins exclusively within DEJ and the adjacent enamel organic matrix (EOM). Our results match 51% of those proteins found in enamel, and 6 of 15 proteins found by Jágr et al. (2019) at the DEJ+EOM. In particular we map COL2A1, DSG1, and F2 in our dataset specifically to the DEJ (Figure 5B). One potential source of discrepancy between the published datasets of De Lima Leite et al. (2018) and Jágr et al. (2019) and our dataset presented here is that they derive from different species. Compared to Jágr et al. (2019), we furthermore use much larger sample volumes for protein extraction, and different instruments and analysis software. In some cases of discrepancy between our dataset and that of Jágr et al. (2019), we locate close homologs or related subunits found in the same protein complexes. Significantly, while we locate the major amelogenesis proteins AMELX, ENAM, AMBN, MMP20, and KLK4 in our enamel dataset and in the spatial pattern expected for each, Jágr et al. (2019) report only AMBN in their enamel proteome dataset and AMELX in their DEJ+EOM protein list; Pandya et al. (2017) report these amelogenesis proteins as western blot data, but report none in their proteomic dataset.

Peptide abundance mapping provides additional clues about protein origin and potential functions within the forming matrix. In this context, our data support the suggestion of previous studies that proteins become entombed between expanding enamel crystals as a result of ameloblast cells shedding proteins and cellular processes during their movement in the context of enamel formation (Goldberg et al., 1998; Smith et al., 2016). The entrapment of proteins in forming enamel is consistent with our observation of highest protein abundance in secretory enamel, and highest peptide diversity in mature enamel, in part due to the amount of enamel matrix protein secretion during secretory stage and the number of cleavage products resulting from successive stages of enamel formation (Figure 1C).

It is important to recognize that microsampling strategies used in proteomic analysis can cause sample cross-contamination of protein and peptides. This can result both from mechanical sampling processes that smear and transport material across adjacent sample areas, from freeze/thaw cycles, or from washing procedures leading to preferential degradation, dispersal, or washing in of organic constituents into adjoining sample areas. These mechanical sampling and washing methods are common techniques in proteomic sampling of enamel (Castiblanco et al., 2015; Charone et al., 2016; Pandya et al., 2017; De Lima Leite et al., 2018; Jágr et al., 2019). Exchange processes that are necessary for the hardening of enamel are facilitated by porosity in maturation stage enamel. This porosity could also increase sensitivity to contamination during sample processing, and might contribute to our observation of increased protein diversity (Robinson et al., 1992, 1994; Brookes et al., 1995; Chen et al., 1995).

Multiple lines of evidence suggest that our proteomic results demonstrate consistency with expected protein distributions. First, in keeping with all well-established models of enamel formation (Smith et al., 2005), our overall protein abundance data (Figure 1C) show highest abundance in secretory and cervical loop regions. This region is the least mineralized in still forming tooth crowns (Bartlett, 2013; Lacruz et al., 2017) and hypsodont teeth which are characterized by their slow growth rate at the cervical margin (Green et al., 2017). Furthermore, our sampling strategy focused on retrieving a sample from the cervical margin, despite tapering enamel thickness in this region (Figure 1A). Therefore, both enamel and dentin are contained in these samples due to the sampling area and this is reflected in their high protein abundance (Figure 1C).

Second, both PCA (Figure 1B) and hierarchical clustering analyses (Figures 2, 3) demonstrate clear spatial segregation of the entire protein dataset into regions known to correspond anatomically to secretion, maturation and eruption. This pattern is further borne out in analysis of specific proteins: AMELX, AMBN, ENAM and MMP20 are found at very high abundances in secretory enamel, at low concentrations thereafter, and at low concentrations in sample blocks known to contain dentin (Figures 1A,D, 4). By contrast and as expected KLK4 is found in maturing and mature enamel but not in secretory enamel, showing that both major enamel proteases are located in their expected distributions (Brookes et al., 1998; Perez et al., 2018).Third, some proteins known to be produced in porcine salivary glands and involved in immune function, such as LCN2, RETN, and PGLYRP1, are found in high abundance and nearly exclusively at the erupted enamel margin (Figure 5B). Similarly, we locate proteins recently reported at the DEJ, specifically, DSG1, FN, and COL2A1, at high abundance at the DEJ in our analyzed teeth (Jágr et al., 2019). Taken together, these data strongly support the integrity of the dataset and the consistency with published results on protein distribution in forming enamel. In addition, while great care was taken during sample preparation to avoid contamination, it is important to recognize that some degree of contamination may affect any proteomic dataset from microsampled tissues.

The confirmation of the presence of DEJ proteins located in this tissue by microsampling previously (Jágr et al., 2019), and the presence of salivary and immune function proteins almost exclusively within erupted enamel (Figure 5B), encourages further study. In particular the location of proteins LCN2, RETN, and PGLYRP1 in erupted enamel raises the question of whether these are being sampled from the enamel pellicle, or may be infiltrating into mature erupted enamel as a normal part of posteruptive enamel transformation (Mamali et al., 2012; Aqrawi et al., 2017; Nylund et al., 2017; Tavladaki et al., 2017; Xiao et al., 2017; Dziarski and Gupta, 2018; Prims et al., 2019).

### Protein Distribution and Enamel Defects

We cross-referenced proteins that we located in secretory enamel with genes associated with enamel defects in the Online Mendelian Inheritance in Man (OMIM) database (Goldberg et al., 2002, 2005; Klingberg et al., 2009; Seymen et al., 2016; Hadjichristou et al., 2017; Porntaveetus et al., 2017). Specifically, we detected neurotensin precursor (NTS), decorin (DCN), collagens III, V, VII and XVII (COL3A1, COL5A1, COL5A2, COL5A3, COL7A1), gap junction protein (GJA1), laminin A3 (LAMA3), laminin C2 (LAMC2), integrin B4 (ITGB), and ACPT (Figures 4, 5 and Supplementary Table 1). While it is unresolved how these affect enamel formation, their mapping onto developmental stages may elucidate possible mechanisms. For example, NTS is a signaling molecule that is upregulated in the plasma of patients with Prader-Willi syndrome, a complex genetic conditions affecting many parts of the body frequently including symptoms of enamel hypoplasia (Butler et al., 2015). Neurotensin is expressed by dentin-forming cells, odontoblasts, possibly to regulate dentinogenesis and nociception, and NTS RNA transcripts have been found in rat enamel organs (Bhatnagar et al., 1995; Moffatt et al., 2006); our spatial analysis associates the neurotensin precursor NTS with the secretory stage of amelogenesis. In contrast, decorin maps onto maturing enamel and consistent with published findings, also bone and dentin. Decorin is a secreted proteoglycan thought to promote extracellular matrix control. *Decorin* null mice show repressed enamel formation in molars and the enamel in incisors lacks the typical structural organization of interwoven bundles of crystallites that is known as prism decussation pattern (Goldberg et al., 2002, 2005; Gubbiotti et al., 2015).

Consistent with previous descriptions of annexin alpha-2 (ANXA2) near Tomes’ processes of ameloblasts and a role in exocytosis and cell sensing during secretory and early maturation stages, we located it to the surface in secretory and early maturation stage enamel (Supplementary Table 1; Bartlett et al., 2006). Both annexin and gelsolin (GSN) help regulate cytoskeletal functions in ameloblast cytotaxis during secretion (Hubbard, 1995). While GSN has been implied in saliva-enamel exchange (Juusela, 2016; Heller et al., 2017), we detected the protein in secretory and early maturation stage enamel, along with cadherin 2 (CDH2) and junction plakoglobin (JUP) (Supplementary Table 1), the latter an important component of ameloblast desmosome complexes (Fausser et al., 1998; Sorkin et al., 2000; Kieffer-Combeau et al., 2001; Verstraeten et al., 2016).

Gap junction protein GJA1 has been suggested to contribute to ameloblast differentiation (Toth et al., 2010) and promotes cell-cell contact and ion flow. Patients with GJA1 mutations suffer from severe enamel hypoplasia with frequent enamel loss prior to, or after eruption (Hadjichristou et al., 2017; Porntaveetus et al., 2017). We detected GJA1 in the middle enamel during early maturation phase (Figure 4). We mapped LAMA3 and LAMC2 within secretory, COL17A1 within maturation, and ITGB4 onto surface enamel. Mutations in LAMA3, LAMC2, COL17A1, ITGA6, and ITGB4 are associated with the blistering skin disorder junctional epidermolysis bullosa and cause severe enamel hypoplasia, grooves and enamel pitting (McGrath et al., 1996; Wright, 2006; Murrell et al., 2007; Almaani et al., 2009; Kim et al., 2013; Wang et al., 2015; Gostynska et al., 2016). Both LAMC2 and LAMA3 are a part of the laminin 332 complex (also called laminin 5), known to be involved in tooth bud differentiation and pre-secretory ameloblast activity, and previously seen observed with amelotin (AMTN) at the interface between ameloblasts and enamel during early and late maturation (Nanci et al., 1993; Yoshiba et al., 2002; Sawada, 2015; Smith et al., 2019). Because we observe LAMA3 at high abundance in secretory enamel, we speculate that this protein may play a role in enamel secretion, possibly related to 332 functions corresponding to cell anchorage, mobility, or signaling (Ryan et al., 1999). High levels of COL7A1 localized to the DEJ, consistent with previous findings, but also in developing enamel indicating continued abundance after the DEJ has formed (McGuire et al., 2014). Mutations in the genes that produce COL3A1, COL5A1 and COL5A2 are associated with Ehlers–Danlos syndrome, which frequently includes hypomineralized and porous enamel (Lloyd et al., 1993; Pope et al., 1996; Burrows et al., 1997; Michalickova et al., 1998; Richards et al., 1998; Takahara et al., 2002; Klingberg et al., 2009). We found COL5A1 and A3 in bone, dentin and at the DEJ, but detected COL3A1 and COL5A2 at low levels in secretory and maturation regions of the enamel (SI 02); previous work has left the status of COL5 in enamel ambiguous (Bronckers et al., 1986).

### Posttranslational Modifications

Our nano-LC-MS/MS proteomic analyses also offered new insights into the spatiotemporal distribution of posttranslational modifications of key proteins during the process of enamel mineralization. Our focus here was on AMELX, which is likely to be phosphorylated at secretion. Consistent with *in vitro* data on the importance of the Ser16 phosphorylation for the stabilization of amorphous calcium phosphate (Wiedemann-Bidlack et al., 2011; Le Norcy et al., 2018), we observed that the ratio of phosphorylated P173+LRAP products to all P173+LRAP products was highest in superficial secretory and early maturation stage enamel (Figure 4A). The spatial pattern seen for the amelogenin P173+LRAP is more complex because we simultaneously track all amelogenin P173+LRAP derived proteins exhibiting the N-terminal sequence (e.g., P173, P56, and their N-terminal derived processing products including P162, P148, P62/3, P45 or TRAP, LRAP, and P40). This pattern was distinct from the distribution seen for the sparse P190 splicing variant products. We detected two candidate molecules that could potentially be responsible for the dephosphorylation: alkaline phosphatase (ALPL) and testicular acid phosphatase (ACPT). While ALPL abundance was low in surface enamel and has been reported previously, ACPT was detected in secretory and early maturation stage enamel, with high abundance in regions where we observe both phosphorylated and unphosphorylated P173+LRAP products (Figure 4A; Pandya et al., 2017). Notably, recent clinical data indicates that ACPT mutations can result in hypoplastic amelogenesis imperfecta (Seymen et al., 2016; Smith et al., 2017). The distributions of ACPT and unphosphorylated P173 plus LRAP in our enamel dataset suggest a potential ACPT-mediated dephosphorylation of AMELX, a hypothesis to be further tested experimentally. This mechanism would support the key role of AMELX phosphorylation *in vivo* for amorphous calcium phosphate stabilization during early stages of enamel formation and the importance of AMELX dephosphorylation for the regulated transition from amorphous to crystalline HAp that is required to complete enamel maturation (Wiedemann-Bidlack et al., 2011; Yamazaki et al., 2017; Le Norcy et al., 2018).

We map an ENAM peptide fragment containing the third known phosphorylation site of ENAM at Ser178 and present in ENAM 89, 142, 155, and 32 kDa cleavage products (Figure 4B). Unlike our quantification of the overall distribution of ENAM which relies on three distinct peptides found within the 89, 142, 155, and 25 kDa cleavage products, we locate the peptide used for quantification of ENAM phosphorylation almost exclusively in mature enamel. This is consistent with the resistance of the 32 kDa fragment to MMP20 proteolysis (Fukae and Tanabe, 1987; Uchida et al., 1991; Tanabe et al., 1994; Fukae et al., 1996; Hu et al., 1997; Al-Hashimi et al., 2009; Brookes et al., 2011). Almost all of the mapped ENAM peptide fragment is phosphorylated, with unphosphorylated forms found in areas where we also see low overall ENAM abundance. We map AMBN Ser17 phosphorylation with a peptide fragment corresponding to the AMBN 15 and 17 kDa N-terminal cleavage products. In contrast, overall AMBN abundance has been mapped using a combination of three peptides found within the N-terminal half of the overall protein. We see that this AMBN peptide is less phosphorylated in immature enamel and near the enamel surface, and more phosphorylated in mature enamel (Figure 4C). Prior immunohistochemical staining has confirmed that N-terminal AMBN cleavage products are present within prism sheath proteins, and in enamel as late as transition stage (Uchida et al., 1997). We hope our findings will spur further investigation into the roles of ENAM and AMBN phosphorylation states within the process of enamel mineralization.

## Conclusion

The diversity of proteins mapped onto the forming porcine tooth crown and mineralizing enamel is striking when compared to the few traditionally invoked amelogenesis proteins. Consistent with past research on the protein contribution to amelogenesis, secretory enamel is a more specialized mineralization space with limited protein diversity compared to more progressed mineralization, such as maturation stage enamel, or fully formed bone and dentin. Further evaluation of proteins unique to secretory enamel compared to all tissues studied here may help to identify fundamental mechanisms of mineralized tissue formation and reveal how particular protein interactions result in the exceptional material properties of enamel. We have here opted for greater spatial resolution at the expense of individual- and tooth-level replicates, and recognize that these results should be further probed in subsequent studies. Nevertheless, our approach to localize and quantify structural enamel matrix proteins and their modifications, specifically, amelogenin phosphorylation during different stages of enamel mineralization provides a basis to validate *in vitro* based models of enamel mineralization. Distinct distributions of other protein groups within the forming enamel matrix provide guidance to unravel protein-mineral interactions and mechanisms of enamel formation. In addition, grouped localization of enamel proteins that facilitate posttranslational modifications advance our understanding of dynamic protein roles in the transient mineralizing matrix. Specifically, we found ACPT in the same secretory enamel region as amelogenin, where amorphous mineral phases are stabilized. Our findings suggest that the mapping of proteomes onto forming mineralizing tissues informs our understanding and guides follow up studies on the mechanisms of tissue growth, etiologies of disease, and bioinspired materials.

## Data Availability

The datasets generated for this study can be found in the PRIDE archive, https://www.ebi.ac.uk/pride/archive/, accession number PXD013604.

## Ethics Statement

All samples were collected from the carcass of a 8-month-old female pig in agreement with IACUC regulations of the Cummings School of Veterinary Medicine, Tufts University and The Forsyth Institute.

## Author Contributions

FB and DG conceived and designed the study. FB, DG, MH, and MP carried out the experimental design for proteomics. FB, DG, and FS prepared the proteome samples for the study. FS and MH conducted the LC-MS/MS measurements and analysis. K-HL and DG wrote the code and conducted the statistical tests. FB, MH, MP, and DG interpreted the data. DG and FB drafted the manuscript. FB, MH, and DG revised the manuscript. All authors approved the final version of the manuscript. FB and MH took responsibility for the integrity of the data analysis.

## Conflict of Interest Statement

The authors declare that the research was conducted in the absence of any commercial or financial relationships that could be construed as a potential conflict of interest.

## Footnotes

1 https://fiji.sc/
