# Supplementary material for: Mapping the Tooth Enamel Proteome and Amelogenin Phosphorylation Onto Mineralizing Porcine Tooth Crowns
Source: Front Physiol. 2019 Jul 30;10:925. doi: 10.3389/fphys.2019.00925 (PMC6682599; doi:10.3389/fphys.2019.00925)
Supplement: Supplementary file 1 [file Data_Sheet_1.PDF]

Supplemental Figure 1: Groups of proteins with similar patterns of abundance changes over the course of mineralization. Patterns of increased and decreased abundance are statistically significant for each protein in each group, as determined by nonparametric ANOVA tests that include a Benjamini-Hochberg correction for multiple analyses.

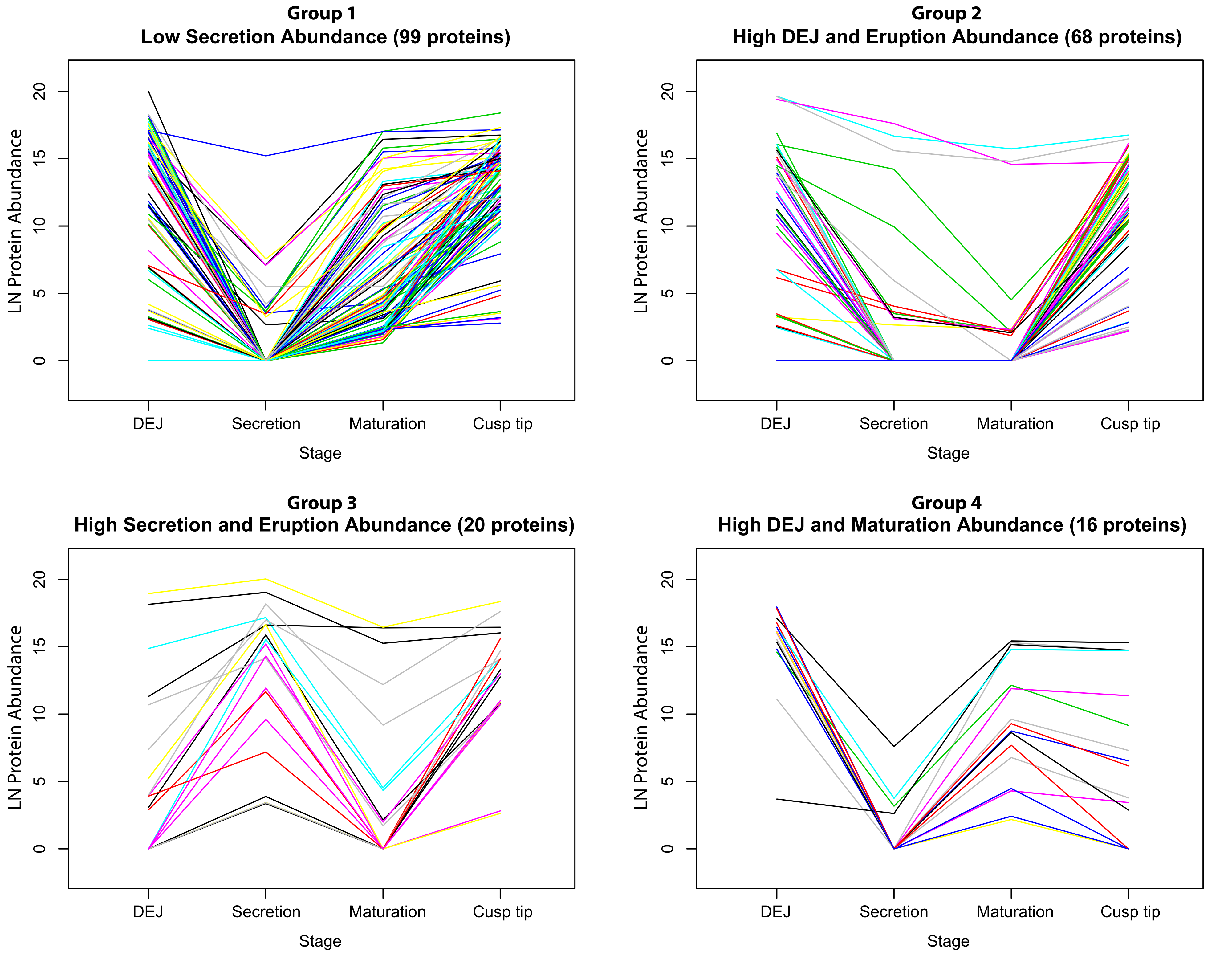

Group 1 Gene abbreviations

NUCB1
ATP6V0A1
CTNND1
LGALS1
MYH14
S100A4
S100A6
ITGAV
SERPIND1
F10
IMMT
SPARC
ACTN4
GNB2L1
VDAC2
COL5A1
MMP2
RPL7A
PHEX
ICA
CANX
HNRNPU
C9
RARRES2
SPP1
PROS
HABP2
FGA
ANXA8
KRT15
VCAN
FSTL1
SERPINE2
HADHA
SERPIN1
KRT3
CORO1A
RPL17
UBA1
TGFBI
SERPINA3-2
OGDH
IGLC
RPS10
CTSL
MYH14
ACO2
ATP6V0D1
RPS3
UQCRC1
LRP1
DNAJC3
CLEC3B
THBS1
MYO6
CALD1
C4BP
CLTC
JCHAIN
DCN
UQCRC2
TXN
PTN
ATP5D
KRT75
NCL
RPS15A
HSPB1
ATP5O
UQCRFS1
COL12A1
HSP90AB1
COL2A1
COL6A1
PRDX2
grp-58
HSPG2
LMNB1
DSP
CD44
PFN1
GOT1
ETFA
AHNAK
HADH
A1BG
RPS8
CRABP1
ATPsynD
RPSA
SERPINA36
ANXA1
PKP1
ANXA1
DSG1
VCP
HRG
TTR
PHB2

Group 2 Gene abbreviations

OGN
P4HA1
SLC25A3
HSPA9
SEMA3E
EMILIN1
PLS1
HP1BP3
RRBP1
MYH9
RPS26
APP
TNC
K6-irs1
CRYAB
CTNNB1U
FKBP7
COL5A3
SCUBE1
RPS13
MMP10
NDUFS3
IGHG
S100A10
SDHA
SET
HNRNPH1
ATP5F1
H2AFY
SLIT3
VIT
COX5B
COL11A1
SPTAN1
POSTN
RPS3A
ANXA4
C5
SLC4A1
LGALS3
PAM
ATP5L
TPP1
MYOF
RPL22
RPL18A
HK1
NDUFS1
NDUFB11
CD248
ALB
PDHB
FLNC
TCN2
RPL13
PLG
TGFBI
COL1A2
TFRC
RPN2
DPYSL2
NPM1
RPL35
MTCH2
DYNCH
TMED10
RPN1
COX5A

Group 3 Gene abbreviations

RPS18
ATP6AP1
RPL15
RPS4X
MMP20
FGA
MYH14
HNRNPA3
Jup
KRT13
AMELX
TNN
RPL12
S100A14
SERPINA38
HIST2B
RPL13A
RPLP0
LAMA3
ANXA2

Group 4 Gene abbreviations

CLEC11A
TKT
TGFB1
TGFB2
IGHG
C3
SCRN1
APOE
CHID1
OLFML3
PCOLCE
SEMA3D
CPB2
SSR1
IGV
FGFR1

Group 5 Gene abbreviations (Low DEJ)

S100A8
TUFM
MB
SRI
RPL9
COL12A1

Group 6 Gene abbreviations (High DEJ)

ECM2
P4HA2
APOA2
COL17A1
SFRP2
SDF4
BGN

Group 7 Gene abbreviations (High secretory)

SERPINC1
APOA1
AMBN
TLN1
DPT

Group 8 Gene abbreviations (High secretion + maturation)

EEF1G
ATP6V1E1
KRT10
